# Barriers and Opportunities for Using Wearable Devices to Increase Physical Activity Among Veterans: Pilot Study

**DOI:** 10.2196/10945

**Published:** 2018-11-06

**Authors:** Rebecca H Kim, Mitesh S Patel

**Affiliations:** 1 Perelman School of Medicine University of Pennsylvania Philadelphia, PA United States; 2 Penn Medicine Nudge Unit University of Pennsylvania Philadelphia, PA United States; 3 Wharton School University of Pennsylvania Philadelphia, PA United States; 4 Crescenz Veterans Affairs Medical Center Philadelphia, PA United States

**Keywords:** veterans, wearable devices, connected health device, physical activity, mobile phone

## Abstract

**Background:**

Few studies have examined the use of wearable devices among the veteran population.

**Objective:**

The objective of this study was to evaluate veterans’ perceptions of and experiences with wearable devices and identify the potential barriers and opportunities to using such devices to increase physical activity levels in this population.

**Methods:**

Veterans able to ambulate with or without assistance completed surveys about their mobile technology use and physical activity levels. They were then given the option of using a wearable device to monitor their activity levels. Follow-up telephone interviews were conducted after 2 months.

**Results:**

A total of 16 veterans were enrolled in this study, and all of them agreed to take home and use the wearable device to monitor their activity levels. At follow-up, 91% (10/11) veterans were still using the device daily. Veterans identified both opportunities and barriers for incorporating these devices into interventions to increase physical activity.

**Conclusions:**

Veterans engaged in using wearable devices at high rates.

## Introduction

Regular physical activity is associated with numerous health benefits, including reduced risk of cardiovascular disease. Simply increasing step count is thought to lower the 10-year risk of death [1]. However, fewer than half of all veterans achieve the recommended physical activity levels to improve their health [2]. Many stakeholders, including the Veterans Health Administration (VHA), are interested in the potential of using mobile technologies such as smartphones and wearable devices to change health behaviors [3]. In particular, there is growing interest in the potential of wearable activity tracking devices to facilitate increased physical activity. These wearable devices provide feedback about physical activity levels in the form of step counts and may encourage people to monitor and change their daily activities. They also offer a reward for positive behavior changes by indicating that the day’s step goals have been achieved. Both techniques can be motivating for people attempting to make behavioral changes [4,5]. Wearable devices often differ from traditional pedometers in both their technology and ability to wirelessly transmit activity data. In 2015, the VHA commissioned a systematic review of the published literature on the use of wearable devices in veteran populations [6]. The review found that 12 of 14 trials focused on using wearables for physical activity but that more evidence was needed on how these technologies impact veterans. The objective of this exploratory study was to evaluate veterans’ perceptions of and experiences with wearable activity trackers to identify barriers and opportunities for their use in a future clinical trial using social interventions to increase physical activity among veterans.

## Methods

### Recruitment

In 2017, we recruited a convenience sample of adult veterans at the Corporal Michael J Crescenz Veterans Affairs Medical Center (CMC VAMC) in Philadelphia using flyers outside of clinic sites. Inclusion criteria were as follows: (1) patients of a CMC VAMC clinic and (2) able to ambulate with or without assistance. Interested veterans were offered US $10 in compensation for their participation.

### Study Design

All participants were asked to complete surveys on sociodemographic characteristics, experiences with and attitudes toward mobile technology (adapted from McInnes et al) [7], and physical activity levels using the short-form of the International Physical Activity Questionnaire [8]. After survey completion, we tested whether veterans would be willing to take home and use a wearable device to monitor their activity levels for 2 months. The Nokia Go wearable device was offered to interested participants because it can be worn on the wrist continuously without need for battery charging. Veterans were given the option of using this device and offered US $15 to complete a 2-month follow-up telephone interview. The interview script focused on device use (prior to the study, currently, and future intentions) and interest in using the device in a future intervention (interest in participation and being paired with a family member, friend, or veteran). The interviewer (RHK) coded responses by question and identified representative quotes of common themes from the interview. Responses and themes were reviewed and adjudicated by a second reviewer (MSP). This study was approved by the CMC VAMC Institutional Review Board.

## Results

The sample comprised 16 veterans with a mean age of 60.6 (SD 12.5) years; 88% (14/16) veterans were males (Table 1). All participants reported having regular access to the internet and a smartphone. In the past week, 12 participants had access to a tablet, but only 3 had access to a connected health device (1 had a smartwatch, 1 had a Bluetooth-connected hearing aid, and 1 had a smart pill bottle). The mean physical activity level was 59.1 (SD 57.6) MET-minutes per week. All 16 veterans agreed to take home and use a wearable device to track their activity. At follow-up, 91% (10/11) veterans were still using it daily, but 1 participant had misplaced it after 6 weeks of daily use.

Overall, participants felt positively about wearable activity tracking devices. Several participants mentioned feeling confident that most veterans they know would benefit greatly from owning a wearable device and cited financial restraints as the primary reason why they had not previously owned one themselves. When asked about potential barriers to using these devices in larger interventions for veterans, one participant stated: “people might not be comfortable with the idea of someone else tracking their behavior” (Table 2).

Many participants reported that the device motivated them to make incremental adjustments to their behavior by providing feedback on their daily activity. One veteran explained, “When I’m out, I tend to look and see if I got a star. If it doesn’t hit the star, I know I didn’t get out much that day […] I might take some more steps if I don’t hit the star.” This activity change was not limited to physical activity. Several participants commented on the utility of the sleep tracking ability. For example, one veteran noted, “it made me set my alarm so I don’t sleep too long. I was sleeping too long.”

When asked about how this feedback translated to behavioral change, however, participants had variable experiences and identified both opportunities and barriers to incorporating wearable activity monitors into interventions to increase physical activity (Table 2). One veteran stated, “this increased my activity 100 percent,” and he credited the device with helping him lose 9 pounds and improve control of his diabetes. However, most of the participants noted that rather than motivating them to meet a minimum level of daily physical activity, the device kept them moving on days spent out of the house. For example, one veteran said, “sometimes I set goals, sometimes I just go with it,” indicating that the device helped him more so when he was already motivated.

Lastly, veterans also expressed contrasting opinions about the potential utility of combining this device with a social incentive to increase physical activity. Several felt that it would be beneficial, and one stated that it “would be motivating and could build on the MOVE! program.” However, 2 veterans noted that a disability might prevent them from participating in such a program. For these participants, the device allowed them to set their own physical activity goals based on their physical abilities.

**Table 1:** Baseline participant characteristics (N=16).

Characteristic		Value
**Gender, n (%)**		
	Female	2 (13)
Age (years), mean (SD)		61.5 (11.6)
**Race or ethnicity, n (%)**		
	Non-Hispanic black	7 (44)
	Non-Hispanic white	7 (44)
	Other	2 (13)
**Marital status, n (%)**		
	Married	6 (38)
	Separated	4 (25)
	Never married	6 (38)
**Education, n (%)**		
	Completed high school or obtained General Education Diploma	8 (50)
	Some college	3 (19)
	Completed college	5 (31)
**Annual household income, n (%)**		
	<US $50,000	9 (56)
	US $50,000-100,000	7 (44)
**Employment status, n (%)**		
	Employed full-time	4 (25)
	Employed part-time	2 (13)
	Not employed or retired	10 (63)
**Living situation, n (%)**		
	Own house or apartment	8 (50)
	Rent house or apartment	6 (38)
	Living with friend or relative	1 (6)
	Living in a shelter	1 (6)
**Military service, n^a^**		
	Between WWII and the Korean Conflict	1
	The Korean Conflict (1950-1955)	1
	Vietnam (1961-1975)	5
	Post-Vietnam	8
	1991-2001	1
	After 2001	3

^a^Some people served during multiple periods.

**Table 2:** Veteran perspectives on opportunities and barriers to using wearable activity monitors to increase physical activity.

Perspective	Opportunity	Barrier
Providing wearable activity monitors to veterans	“Veterans would like it because devices like that are very expensive.”	“People might not be comfortable with the idea of someone else tracking their behavior.”
Efficacy of wearable activity monitors in creating a behavioral change	“This increased my activity 100%. I lost 9 lbs. I went from taking three meds [for diabetes] to one, and I didn't have to go on insulin.” “When I'm out I might take some more steps if I don't hit the star” “I can check when walking, sleeping. It allowed me to adjust my behavior. It made me set my alarm so I don't sleep too long. I was sleeping too long.”	“When I’m out and about, I set the goals differently. Sometimes I set goals, sometimes I just go with it.”
Potential interventions combining the device with a social incentive	“Sometimes you need another person.” “I think [it would help]. I didn’t think I’d get into the watch, but I did, so yes.” “[Being paired with another person] would be motivating and could build on the MOVE! program [which is a national weight management program designed by the Veterans Affairs National Center].”	“Hypothetically I would [be willing to be paired with a partner], but my disability prevents me from doing certain things, so it would be challenging.” “I do better alone.”

## Discussion

### Principal Findings

In this pilot study, there were several findings that could help inform future research on using wearables to help veterans increase physical activity. First, veterans engaged in using wearable activity trackers at high rates. This was the case even though most veterans in our sample had limited prior experience with these devices. Second, population-based studies suggest that wearable activity tracking devices are more likely to be used by individuals who are females, younger, and already very physically active [9]. However, our findings suggest that if barriers to access of wearable activity tracking devices are reduced, these technologies may play a meaningful role in increasing physical activity levels among the older veteran population. Of note, several veterans mentioned that they felt other veterans in their social network would benefit from activity tracking devices.

Third, veterans felt that the wearable devices were useful for monitoring their physical activity levels, but similar to prior work [3,10], more could be done to help motivate them. For example, many veterans were interested in how these devices could be paired with use among their social networks in ways that incentivized them to increase their activity. Future research is needed to identify the social support that would most effectively supplement the use of these devices to increase physical activity in veterans. Fourth, several participants also commented on the utility of the sleep tracking function, suggesting that this functionality could be useful to incorporate in the design of future interventions [11].

### Limitations

Our findings are limited by a small sample size from a single Veterans Affairs facility. However, this is one of the first evaluations of its kind among veterans and suggests that if programs are well designed, these devices could play a meaningful role in helping veterans change their physical activity behavior.

### Comparison With Prior Work

Despite the growing body of literature on the potential of wearable activity tracking devices, few studies have explored their potential use among veterans.

### Conclusions

Wearable activity tracking devices have the potential to be used in interventions targeting increased physical activity levels in veterans.

## Abbreviations

CMC VAMC: Corporal Michael J Crescenz Veterans Affairs Medical Center
VHA: Veterans Health Administration
